# Chronic developmental hypoxia alters mitochondrial oxidative capacity and reactive oxygen species production in the fetal rat heart in a sex‐dependent manner

**DOI:** 10.1111/jpi.12821

**Published:** 2022-08-17

**Authors:** Kerri L. M. Smith, Agnieszka Swiderska, Mitchell C. Lock, Lucia Graham, Wulan Iswari, Tashi Choudhary, Donna Thomas, Hager M. Kowash, Michelle Desforges, Elizabeth C. Cottrell, Andrew W. Trafford, Dino A. Giussani, Gina L. J. Galli

**Affiliations:** ^1^ Division of Cardiovascular Sciences, School of Medical Sciences, Faculty of Biology, Medicine and Health University of Manchester Manchester UK; ^2^ Department of Physiology Development and Neuroscience University of Cambridge Cambridge UK

**Keywords:** fetal, heart, hypoxia, melatonin, metabolism, mitochondria, ROS

## Abstract

Insufficient oxygen supply (hypoxia) during fetal development leads to cardiac remodeling and a predisposition to cardiovascular disease in later life. Previous work has shown hypoxia causes oxidative stress in the fetal heart and alters the activity and expression of mitochondrial proteins in a sex‐dependent manner. However, the functional effects of these modifications on mitochondrial respiration remain unknown. Furthermore, while maternal antioxidant treatments are emerging as a promising new strategy to protect the hypoxic fetus, whether these treatments convey similar protection to cardiac mitochondria in the male or female fetus has not been investigated. Therefore, using an established rat model, we measured the sex‐dependent effects of gestational hypoxia and maternal melatonin treatment on fetal cardiac mitochondrial respiration, reactive oxygen species (ROS) production, and lipid peroxidation. Pregnant Wistar rats were subjected to normoxia or hypoxia (13% oxygen) during gestational days (GDs) 6–20 (term ~22 days) with or without melatonin treatment (5 µg/ml in maternal drinking water). On GD 20, mitochondrial aerobic respiration and H_2_O_2_ production were measured in fetal heart tissue, together with lipid peroxidation and citrate synthase (CS) activity. Gestational hypoxia reduced maternal body weight gain (*p* < .01) and increased placental weight (*p* < .05) but had no effect on fetal weight or litter size. Cardiac mitochondria from male but not female fetuses of hypoxic pregnancy had reduced respiratory capacity at Complex II (CII) (*p* < .05), and an increase in H_2_O_2_ production/O_2_ consumption (*p* < .05) without any changes in lipid peroxidation. CS activity was also unchanged in both sexes. Despite maternal melatonin treatment increasing maternal and fetal plasma melatonin concentration (*p* < .001), melatonin treatment had no effect on any of the mitochondrial parameters investigated. To conclude, we show that gestational hypoxia leads to ROS generation from the mitochondrial electron transport chain and affects fetal cardiac mitochondrial respiration in a sex‐dependent manner. We also show that maternal melatonin treatment had no effect on these relationships, which has implications for the development of future therapies for hypoxic pregnancies.

## 1 INTRODUCTION

The growth and survival of the developing fetus is critically dependent on optimal maternal health. Fluctuations in the intrauterine environment can be detrimental to the fetus and can permanently alter the functional capacity of vital organs.^1^ In particular, insufficient fetal oxygen supply (hypoxia) is a common pregnancy complication that occurs in a wide range of conditions, including pre‐eclampsia, placental insufficiency, and high‐altitude pregnancies.^1^ In the short term, hypoxia causes a redistribution of fetal blood flow to hypoxia‐sensitive organs, such as the fetal heart.^2^, ^3^ However, in the longer term, gestational hypoxia can overwhelm this circulatory protection, and lead to impaired cardiac development,^4^, ^5^, ^6^ thickening of the aortic wall,^7^ and reduced cardiac output in the fetus.^8^ Offspring of hypoxic pregnancies display cardiac abnormalities in adulthood and appear to be sensitized to ischemia/reperfusion injury.^7^, ^9^, ^10^, ^11^, ^12^, ^13^, ^14^, ^15^, ^16^, ^17^ These findings support epidemiological evidence from human pregnancies, which show hypoxia during development can increase the risk of cardiac dysfunction in offspring.^18^, ^19^, ^20^, ^21^, ^22^, ^23^ Combined, evidence derived from human clinical studies and from experimental preclinical models strongly supports that fetal hypoxia is an independent risk factor for offspring cardiovascular disease. Understanding the factors that drive cardiac dysfunction in hypoxic pregnancies provides an exciting opportunity to develop therapeutics that prevent abnormal developmental programming of cardiovascular disease.

Cellular dysfunction in hypoxia mainly occurs because oxygen is vital for the production of ATP by oxidative phosphorylation (OXPHOS). Briefly, electrons derived from carbohydrates, fats, and proteins are transferred to the mitochondrial electron transport chain, where they move through a series of complexes and finally bind to oxygen at Complex IV (CIV). The movement of electrons provides the energy for CI, CIII, and CIV to pump protons against their electrochemical gradient and establish a proton‐motive force that drives ATP production through complex V (the F_1_F_0_ ATP‐synthase).^24^ Although most electrons are transported to CIV, a small proportion slip from the chain and bind directly to molecular oxygen to produce superoxide.^25^ When oxygen becomes limiting, the electron transport chain is inhibited and electron slip becomes more common, leading to reduced ATP production and the overproduction of reactive oxygen species (ROS).^26^ Consequently, prolonged periods of hypoxia are associated with energy depletion and oxidative stress, which can ultimately lead to cell death. Nevertheless, studies on adult mammals have shown mitochondria are plastic organelles that are able to adapt and remodel in hypoxic environments. For example, acclimatization to high‐altitude hypoxia is commonly associated with a reduction in mitochondrial content, a downregulation of enzymes involved in OXPHOS and an upregulation of anaerobic pathways.^27^, ^28^ While the strategy is thought to limit ROS production and oxidative stress, these modifications will reduce ATP production, which can have adverse consequences for cellular survival.^27^ Although these processes are relatively well‐studied in the adult mammalian heart, far less is known about the adaptive capacity of fetal mitochondria. It is important to fill this knowledge gap, because adaptive responses during early life can often be permanent, leading to life‐long changes in morphology, physiology, and behavior.^29^

During normal fetal development, a metabolic shift from anaerobic to aerobic respiration occurs during mid‐late gestation and the heart relies on OXPHOS for the majority of ATP production.^30^ Therefore, hypoxia is expected to significantly impact ATP production and ROS generation, particularly during the latter stages of fetal development. Indeed, work from our laboratory^31^ and others^32^, ^33^ has shown that adult offspring from hypoxic pregnancies have sex‐dependent alterations in mitochondrial respiration, protein expression, enzymatic activity, and ROS production, suggesting that hypoxia programs mitochondrial pathways during fetal development. In support of this notion, previous work in guinea pigs has shown that late gestational hypoxia reduces protein expression of CI, CIII, and CV in male and female fetal hearts, as well as a reduction in CIV activity in the male fetal heart.^34^ Similar to adult mammals, this response may represent a compensatory adjustment to limit mitochondrial ROS production by reducing respiration rates. However, the reduction in CI protein expression was not associated with lower enzymatic activity, and another study using the same model showed evidence of lipid peroxidation, suggesting that the overproduction of ROS persists despite these adaptations.^35^ To better understand the functional effects of fetal hypoxia on fetal cardiac mitochondria and their adaptive significance, it is important to measure cardiac mitochondrial respiration and ROS production.

In addition to studying the mechanisms that drive fetal cardiac dysfunction in hypoxia, it is essential to develop an effective therapeutic strategy to prevent cardiovascular disease in adulthood. In this respect, maternal antioxidant treatment is emerging as a promising new approach. Several studies have shown that antioxidants can prevent fetal cardiac remodeling and dysfunction in adulthood, such as maternal treatment with vitamin C, allopurinol, statins, MitoQ, and melatonin.^9^, ^11^, ^36^, ^37^, ^38^, ^39^, ^40^, ^41^ Antioxidant protection on the offspring of hypoxic pregnancy manifests in several ways, including enhanced antioxidant capacity, improvements in transplacental oxygenation, diminished endothelial dysfunction, and normalization of arterial blood pressure.^38^ Interestingly, the protective effects are enhanced if mitochondria‐targeted antioxidants, such as MitoQ, are used,^39^ suggesting ROS production from the mitochondria may play a significant role in programming cardiac dysfunction. Despite the promise of antioxidants in treating hypoxic pregnancies, very little is known about the effects of these compounds on fetal mitochondrial function and ROS production, even in healthy pregnancy. In addition, no study has investigated the sex‐dependent impact of developmental hypoxia on fetal cardiac mitochondrial respiration, nor the impact of maternal antioxidant treatment. This information is essential to assess the suitability of antioxidants as therapeutic agents in complicated pregnancies.

Therefore, the first objective of the present study was to determine the sex‐dependent effects of developmental hypoxia on fetal cardiac mitochondrial respiratory capacity, ROS production, and lipid peroxidation. The second objective was to assess the suitability of maternal melatonin treatment as a therapeutic intervention for fetal hypoxia. We have chosen melatonin as our treatment because it is already safely consumed by people to avoid jetlag,^42^ it can be transported across the mitochondrial membrane and it is also synthesized in the matrix,^43^ so it is considered a mitochondria‐targeted antioxidant.^44^, ^45^, ^46^ Furthermore, melatonin acts at multiple levels in the mitochondria by upregulating antioxidant enzymes, increasing the activity of respiratory complexes, and directly scavenging free radicals.^45^, ^47^, ^48^ Importantly, recent studies have shown that treatment of hypoxic pregnancies with melatonin prevented all alterations in cardiovascular structure and function in adult offspring.^11^ Melatonin is already being used in clinical trials for complicated pregnancies,^49^, ^50^, ^51^ and can readily cross the placenta.^11^ However, nothing is known about the effects of melatonin on fetal cardiac mitochondrial function.

## 2 MATERIALS AND METHODS

### 2.1 Animals

All procedures were carried out in accordance with The Animals (Scientific Procedures) Act, 1986. The ARRIVE guidelines were followed for reporting the use of animals in scientific experiments.^52^ Local ethical approval was granted by The University of Manchester Animal Welfare Ethical and Review Board. Female Wistar rats (250–275 g) were purchased from Charles River, and maintained at the University of Manchester. All animals were allowed to acclimatize for at least 1 week upon arrival before use. Animals were group‐housed in individually ventilated cages under standard conditions, with a regular 12:12 h light/dark cycle. Access to food (standard rat chow, Envigo) and water was provided ad libitum.

### 2.2 Hypoxia model

**Figure 1:**
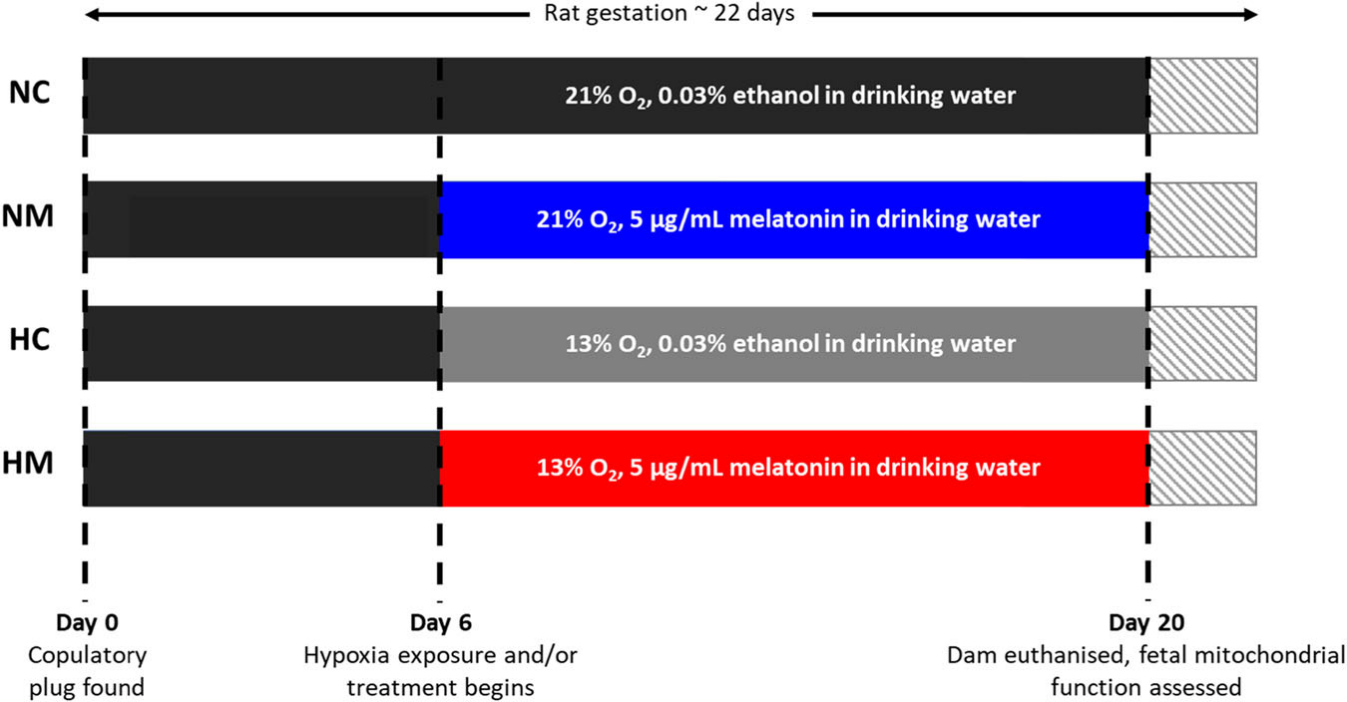
Timeline of hypoxia exposure and melatonin treatment during rat fetal development. Term rat gestation occurs around 22 days. HC, hypoxia control; HM, hypoxia melatonin; NC, normoxia control; NM, normoxia melatonin.

Following acclimatization, dams were time‐mated overnight with Wistar males, with the presence of a copulatory plug the following morning used to confirm pregnancy. The date the plug was found was taken as the GD 0. On GD 6, pregnant rats were randomly assigned into four different groups: normoxic control (NC, *n* = 9), normoxic melatonin (NM, *n* = 11), hypoxic control (HC, *n* = 11), and hypoxic melatonin (HM, *n* = 8), and from this point onwards were individually housed. For hypoxic exposure, rats were transferred to an environmental chamber (Coy O2 In Vivo Glove Box, Coy Laboratory Products) at GD 6 where they were subjected to 13 ± 0.1% oxygen (O_2_). For antioxidant treatment, the drinking water of NM and HM animals was supplemented with 5 µg/ml melatonin (Sigma Aldrich) dissolved in the minimum required volume of ethanol (final concentration of ethanol, 0.03%). An equal concentration of ethanol was added to NC and HC drinking water as vehicle control. The dose of melatonin was chosen as it is comparable with the maximal effective dose recommended for overcoming jet lag in humans.^42^ Melatonin was made up fresh every other day and water bottles were covered to prevent light‐induced breakdown. Maternal food and water intake were regularly measured throughout pregnancy, and maternal body weight was measured before and at the end of the exposure. On GD 20, dams were killed by cervical dislocation, decapitated, and maternal trunk blood was collected. The uterus was externalized, and fetuses and associated placentae were individually weighed and recorded. Fetal trunk blood was collected and pooled per litter. Maternal and fetal blood was collected in heparinized tubes and centrifuged at 5000 rpm for 5 min, aliquoted, and frozen at −80°C for subsequent analysis of plasma concentration of melatonin. From each litter, two hearts were used for mitochondrial analysis in separate chambers (one heart per chamber) and two hearts were snap frozen in liquid nitrogen and stored at −80°C for future analysis. For each set of experiments, pups were used from the same position on the uterine horn to account for the potential differential exposure to hypoxia within the uterus.^12^ Tail tips were collected and stored at −20°C for sex determination by SRY genotyping. A schematic of the hypoxia model is shown in Figure 1.

### 2.3 SRY genotyping

Tail tips were thawed and DNA extracted using RED Extract‐N‐Amp Tissue polymerase chain reaction (PCR) kit, according to the manufacturer's instructions. PCR was performed in tubes containing 0.5 μl of extracted DNA, and 19.5 μl of a master mixture (1× Red *Taq* DNA Polymerase Master Mix (VWR International)), 0.2 μM of each primer (Table 1).^53^ PCR conditions were denaturation at 94°C for 4 min, followed by 36 cycles of amplification at 94°C for 1 min, 52°C for 1 min and 72°C for 1 min, and then extension at 72°C for 10 min with a thermocycler (C1000 Touch™; BioRad). For analysis of PCR products, each PCR mixture was subject to electrophoresis in a 2% agarose gel containing SafeView (Applied Biological Materials) for 40 min at 90 V. Gels were visualized using a ChemiDoc™ (MP; BioRad). The presence of an SRY band in addition to the positive control band for beta‐actin was used to confirm sex as male.

**Table 1:** Primers for SRY genotyping

Gene	Forward primer sequence	Reverse primer sequence
*Sry*	5′–TACAGCCTGAGGACATATTA–3′	5′–GCACTTTAACCCTTCGATGA–3′
*Actb*	5′–AGCCATGTACGTAGCCATCC–3′	5′–TGTGGTGGTGAAGCTGTAGC–3′

*Note*: Sequences for forward and reverse *Sry* and *Actb* primers. *Actb* was included as a control for DNA quality.

### 2.4 Melatonin assay

The concentration of maternal and fetal melatonin levels in plasma was determined in all samples within one assay/standard curve. Melatonin levels were determined using a colorimetric melatonin ELISA kit (code ‐ ab283259; Abcam). The sensitivity of the assay was 2.5 pg/ml.

### 2.5 Mitochondrial analysis

Hearts were excised from pups, homogenized in ice‐cold MIRO5 buffer (ethylene glycol‐bis(ß‐aminoethyl ether)‐N,N,N',N'‐tetraacetic aicd  0.5 mM, MgCl_2_ 3 mM, K‐MES 60 mM, KH_2_PO_4_ 10 mM, 4‐(2‐hydroxyethyl)‐1‐piperazineethanesulfonic acid 20 mM, sucrose 110 mM, and 0.1% bovine serum albumin (BSA)) and filtered through a nylon mesh (200 µm filter). Homogenate (60 µl) was quickly (within 10 min) injected into an Oroboros Oxygraph‐O2K (Oroboros Instruments) for simultaneous measurement of mitochondrial respiration and H_2_O_2_ production. A substrate–uncoupler–inhibitor–titration (SUIT) protocol was utilized (see details below) to examine oxygen consumption and ROS production at different electron transport chain complexes (Supporting information: Figure 1). Results were normalized to protein content (90 ± 3 µg), determined using the Bradford method, and corrected for the BSA content of the buffer (Bio‐Rad Laboratories). Remaining homogenate was frozen at −80°C for determination of CS activity.

### 2.6 Mitochondrial oxygen consumption and ROS production

Production of H_2_O_2_ was measured through the addition of Amplex® UltraRed (10 μM), 1 U/ml horseradish peroxidase (HRP), and 5 U/ml superoxide dismutase (SOD) to each chamber, before addition of homogenate. Amplex® UltraRed oxidizes in the presence of H_2_O_2_ to form resorufin, using HRP as a catalyst, while SOD converts extramitochondrial superoxide (O_2_−) to H_2_O_2_. Amplex® UltraRed was excited at 563 nm and emission measured at 587 nm. An H_2_O_2_ calibration was carried out once in each experiment by adding two additions of 40 µM H_2_O_2_ to achieve a chamber concentration of 0.1 and 0.2 µM. CI substrates pyruvate (6.25 mM), malate (2 mM), and glutamate (10 mM) were then added to each chamber, followed by the homogenate (90 ± 3 µg), to achieve LEAK respiratory state with CI substrates in the absence of adenylates (LEAK_CI_). Saturating ADP (5 mM) was then added to activate OXPHOS with CI substrates (OXPHOS_CI_). This was followed by succinate addition (10 mM), to measure the additive effects of CII substrates on OXPHOS (OXPHOS_CI + CII_). To assess maximum electron transfer capacity (ETC_CI + II_), mitochondria were uncoupled through the addition of carbonyl cyanide‐4‐(trifluoromethoxy)phenylhydrazone (FCCP), which was titrated in steps to a final concentration of 0.75–3 µM, until additions failed to cause a subsequent increase in respiration. Following this, the CI inhibitor rotenone (0.15 μM) was added to assess ETC_CII_ with CII substrates only. Next, CIII inhibitor antimycin A (12.5μM) was added to block the electron transport pathway so residual nonmitochondrial oxygen consumption (ROX) could be measured. To measure CIV activity, the electron donor N,N,N′,N′‐tetramethyl‐p‐phenylenediamine (TMPD, 0.5mM) was added, in combination with ascorbate (2 mM) to prevent autoxidation of TMPD. Finally, the CIV inhibitor sodium azide (50 mM) was added, to measure background nonmitochondrial oxygen consumption following the addition of TMPD.

### 2.7 Lipid peroxidation assay

Lipid peroxidation was assessed using the TBARS (thiobarbituric acid [TBA] reactive substance) assay, which measures the levels of malondialdehyde (MDA) in biological samples. Frozen hearts were briefly thawed and placed in ice‐cold 10 mM phosphate buffer (pH 7.2), containing 200 μM butylated hydroxytoluene to prevent homogenization‐induced artificial oxidative stress. Hearts were homogenized at 4°C and protein concentration was measured using the Bradford method. Homogenate was added to a TBA‐TCA‐HCl mix (0.375% TBA, 15% trichloroacetic acid [TCA], 0.25 N hydrochloric acid [HCl]) at a 1:2 ratio and incubated at 95°C for 30 min. Samples were then incubated on ice for 10 min, before the sample was added to 1‐butanol at a 2:1 ratio and centrifuged at 15 000*g* for 3min. The top layer containing the malondialdehyde‐thiobarbituric acid  adduct was pipetted into a microplate in duplicate, and absorbance measured at 532 nm. MDA concentrations were determined by using 1,1,3,3‐tetraethoxypropane as a standard, normalized to protein concentration, and expressed as nmoles/mg protein.

### 2.8 CS activity

CS activity was measured using a spectrophotometric method. Frozen samples were thawed and diluted in CS assay buffer (100μM 5,5′‐dithiobis‐(2‐nitrobenzoic acid) [DTNB], 300 μM acetyl coenzyme‐A, 100 mM Tris, 0.1% Triton‐X‐100). Oxaloacetate (0.5 mM) was added, and absorbance was measured repeatedly for 10 min at 412 nm to determine *V*_max_.

### 2.9 Statistics

Fetal and placental weights were pooled to obtain litter averages. For maternal and fetal parameters, a two‐way analysis of variance (ANOVA) test was used with Tukey post‐hoc test (GraphPad Prism 8.1.2). For food and water intake, the two factors were the experimental group and GD. For maternal weight, fetal weight, placental weight, and relative placental weight, the two factors were treatment (hypoxic or normoxic) and drug (control or melatonin). For mitochondrial analysis, lipid peroxidation, and CS activity, multiple measurements were taken from the same litter. Therefore, linear mixed modeling (SPSS Statistics, IBM) was performed to account for the nested (clustered) design of the experiment, for example, observations from different pups from the same dam (with treatment [hypoxic or normoxic] and drug [control or melatonin] as cofactors). A Bonferroni post‐hoc test was used. A multivariate ANOVA (MANOVA) analysis was also performed on respiration and H_2_O_2_ production (treatment [hypoxic or normoxic] and drug [control or melatonin], and the interaction between treatment and drug). The significance of the interaction and the main effects were examined using the Wilks' Lambda statistic. For mitochondrial analysis, lipid peroxidation, and CS activity, data were log‐transformed to achieve normal distribution before statistical analysis. A *p* < .05 was considered significant and *p* < .1 was included on graphs and within the results section for clarity.

## 3 RESULTS

### 3.1 Fetal and maternal parameters

**Figure 2:**
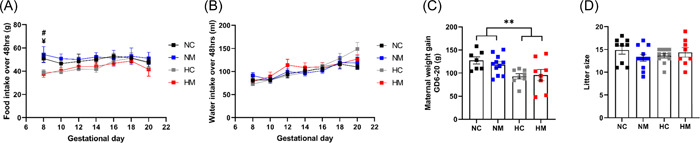
Maternal parameters for dams (gestational day [GD] 6–20). Food and water were monitored every 48 h in normoxic control, normoxic melatonin, hypoxic control, and hypoxic melatonin treated dams (A, B). Maternal weight was measured at GD 6 and 20, and the weights were used to calculate weight gain (C). Litter size was measured following euthanasia at GD 20 (D). *n* = 9 (NC), 11 (NM), 11 (HC), 8 (HM). For food intake, ^#^Significant difference between NM and HC, ^¥^significant difference between NM and HM (*p* < .05). **p < .01 to represent significant effects of hypoxia. Significance was assessed using a two‐way ANOVA. Error bars show mean ± SEM. ANOVA, analysis of variance; GD, gestational day; HC, hypoxia control; HM, hypoxia melatonin; NC, normoxia control; NM, normoxia melatonin.

**Figure 3:**
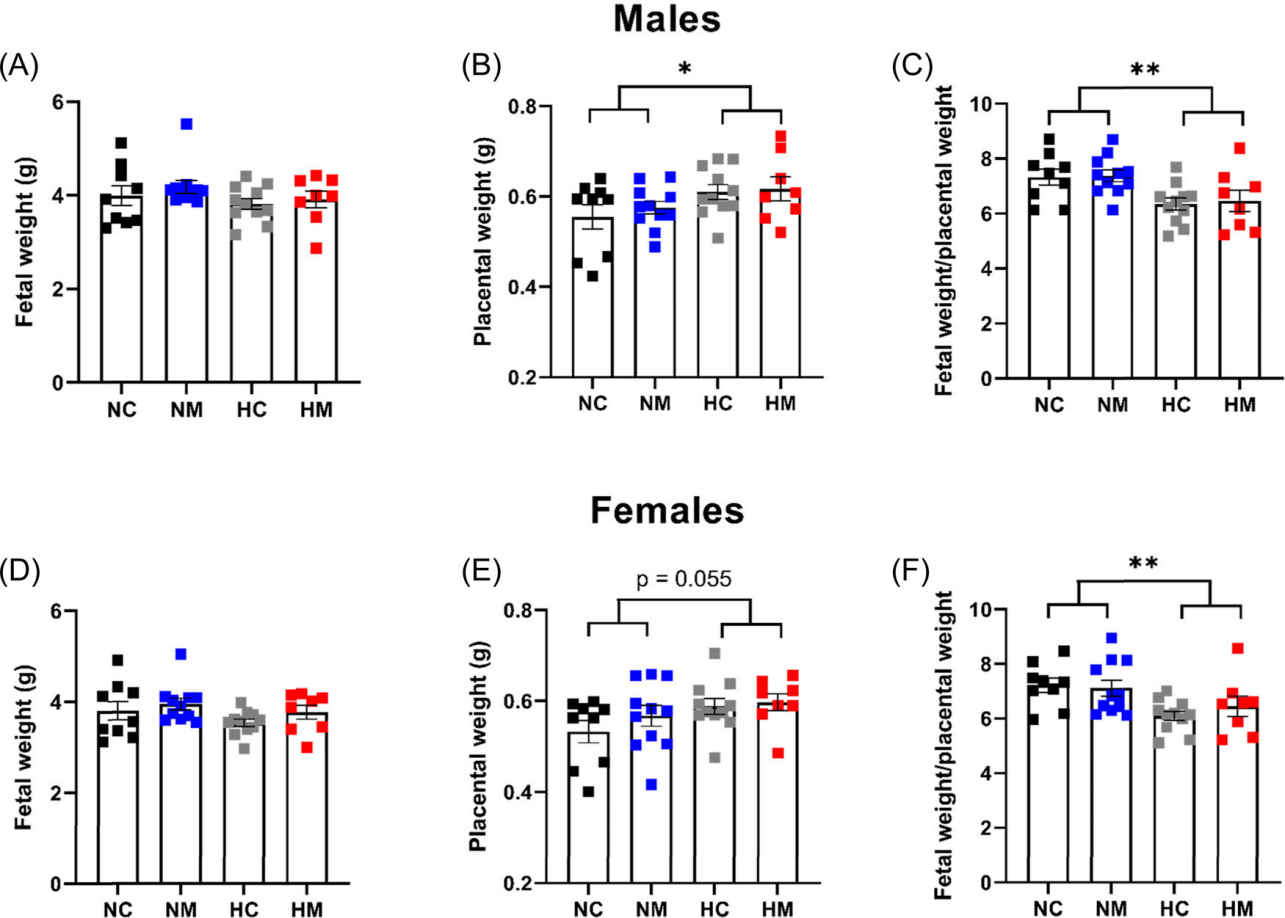
Fetal parameters at GD 20. Fetal weight and placental weight were measured for male fetuses (A–C) and female fetuses (D–F) at GD 20. Data points represent litter averages. *n* = 134 pups from 9 litters (NC), 145 pups from 11 litters (NM), 150 pups from 11 litters (HC), 115 and pups from 8 litters (HM). *p< .05, **p< .01 to represent significant effects of hypoxia. Significance was assessed using a two‐way ANOVA. Error bars show mean ± SEM. ANOVA, analysis of variance; GD, gestational day; HC, hypoxia control; HM, hypoxia melatonin; NC, normoxia control; NM, normoxia melatonin.

Hypoxia did not affect maternal food intake across the hypoxic incubation when compared to the NC group (Figure 2A). However, there was a reduction in maternal food intake at the start of the exposure in both hypoxic groups, compared to the NM group (interaction effect *p* = .027 [hypoxia control vs. normoxia melatonin], *p* = .024 [hypoxia melatonin vs. normoxia melatonin]), which may have reduced maternal weight gain at GD 20 (Figure 2C). However, maternal food intake recovered after 4 days of hypoxia (Figure 2A), and no difference was observed in water intake between the four groups (Figure 2B). While maternal body weight gain during GD 6–20 was lower in the hypoxic groups (main hypoxia effect *p* = .003), there were no differences in litter size or fetal body weight (Figures 2C,D and 3A,D). Hypoxia increased placental weight in both male and female fetuses, but this effect only reached significance in males (main hypoxia effect *p* = .026 [males], *p* = .055 [females]) (Figure 3B,E). Additionally, hypoxia decreased the ratio of fetal weight to placental weight, for both males and females (main hypoxia effect *p* = .001 and .003, respectively) (Figure 3C,F).

As expected, maternal treatment with melatonin increased the concentration of melatonin at GD 20 in both maternal and fetal plasma compared with untreated pregnancies (main melatonin effect *p* < .0001), and hypoxia did not affect fetal melatonin concentrations (Table 2). It should be noted that maternal melatonin concentrations were slightly higher in hypoxic dams compared to normoxic (interaction effect *p* = .081 [hypoxia melatonin vs. normoxia melatonin]). Nevertheless, maternal melatonin treatment had no effect on any of the measured fetal and maternal parameters (Figures 2 and 3).

**Table 2:** Plasma melatonin concentrations

	NC	NM	HC	HM
Maternal plamsa (melatonin) (pg/ml)	**80** ± **13.7**	**515** ± **106.5** ***	**92.9** ± **17.1**	**800.7** ± **118.1** ***
Fetal plasma (melatonin) (pg/ml)	**33.2** ± **4.5**	**308.1** ± **50.7** ***	**57.6** ± **11.8**	**326.9** ± **51.3** ***

*Note*: Values are mean ± SEM for the concentration of melatonin in plasma collected on gestational day 20 from NC, NM, HC, and HM dams and fetuses. *n* = 9–10 per group.

Abbreviations: ANOVA, analysis of variance; NC, normoxic control; NM, normoxic melatonin; HC, hypoxic control; HM, hypoxic melatonin.

*** *p* < .0001 significant effect of melatonin (two‐way ANOVA).

### 3.2 Mitochondrial respiration and CS activity

**Figure 4:**
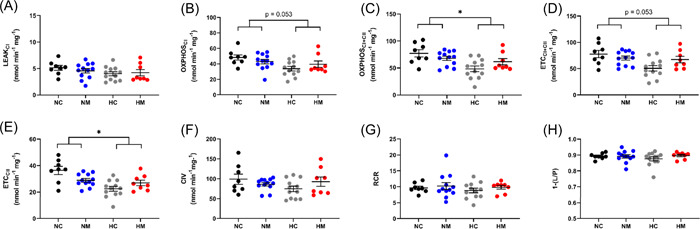
Developmental hypoxia reduces oxidative capacity in heart mitochondria from male fetuses at multiple points of the electron transport chain. Mitochondrial oxidative capacity was measured following a SUIT protocol. Each panel represents a different respiratory state. RCR values (G) and coupling efficiencies (H) were high for all groups, demonstrating mitochondrial homogenate preparations were of good quality. *n* = 8 (NC from 6 litters), 12 (NM from 7 litters), 12 (HC from 7 litters), 8 (HM from 6 litters). *p< .05 (effects of hypoxia). Significance was assessed using a linear mixed model (nested). Error bars show mean ± SEM. 1‐(L/P), coupling efficiency; CI, complex I; CII, complex II; CIV, complex IV; ETC, electron transport capacity; HC, hypoxia control; HM, hypoxia melatonin; NC, normoxia control; NM, normoxia melatonin; OXPHOS, oxidative phosphorylation; RCR, respiratory control ratio; SUIT, substrate–uncoupler–inhibitor–titration.

Mitochondrial homogenate preparations were of high quality, as attested by high respiratory control ratios (9.5 ± 0.4) and OXPHOS coupling efficiency ratios (0.88 ± 0.01), with CI substrates (malate, pyruvate, and glutamate) (Figure 4G,H). Homogenates responded to SUIT chemicals as expected^54^ (Supporting Information: Figure 1).

**Figure 5:**
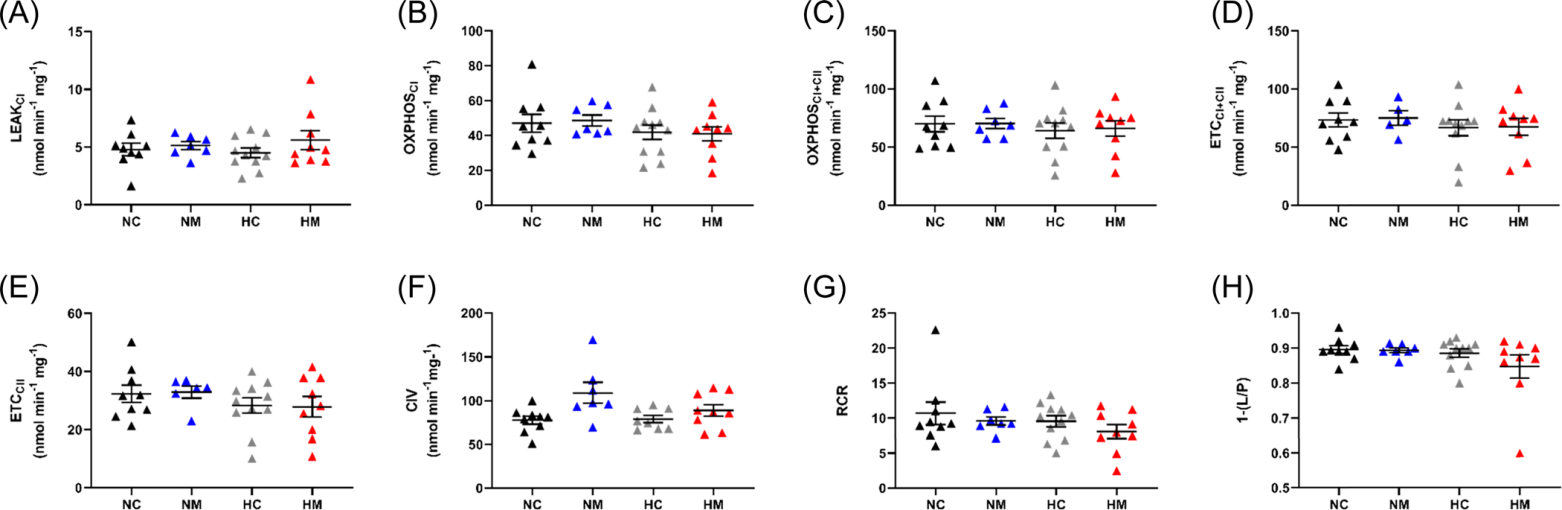
Developmental hypoxia does not change oxidative capacity in heart mitochondria from female fetuses. Mitochondrial oxidative capacity was measured following a SUIT (substrate‐uncoupler‐inhibitor‐titration) protocol. Each panel represents a different respiratory state. RCR values (G) and coupling efficiencies (H) were high for all groups, demonstrating mitochondrial homogenate preparations were of good quality. *n* = 9 (NC from 7 litters), 7 (NM from 5 litters), 11 (HC from 8 litters), 9 (HM from 7 litters). Significance was assessed using a linear mixed model (nested). Error bars show mean ± SEM. 1‐(L/P), coupling efficiency; CI, complex I; CII, complex II; CIV, complex IV; ETC, electron transport capacity; HC, hypoxia control; HM, hypoxia melatonin; NC, normoxia control; NM, normoxia melatonin; OXPHOS, oxidative phosphorylation; RCR, respiratory control ratio; SUIT, substrate–uncoupler–inhibitor–titration.

In heart homogenates from male fetuses, chronic hypoxia caused a decrease in mitochondrial oxygen consumption in the OXPHOX_CI + CII_ and ETC_CII_ states (main hypoxia effect *p* = .031 and .037, respectively) (Figure 4C,E). This effect was also evident in the OXPHOS_CI_ and ETC_CI + CII_ states, but did not reach significance (main hypoxia effect *p* = .053) (Figure 4B,D). All the other respiratory parameters in males (LEAK, CIV respiration, RCR, and OXPHOS‐coupling efficiency) were unaffected by hypoxia (Figure 4A,F,G,H). In contrast to males, there was no effect of hypoxia on any mitochondrial respiratory parameter in female fetuses (Figure 5), with all four groups showing comparable respiration across respiratory states. In both sexes, maternal melatonin treatment had no effects on mitochondrial oxygen consumption in normoxic or hypoxic pregnancies (Figures 4, 5). We also measured enzymatic activity of CS activity as a common marker for mitochondrial content and found no differences between any of the experimental groups in males or females (Figure 8A,C).

### 3.3 Mitochondrial ROS production and oxidative stress

**Figure 6:**
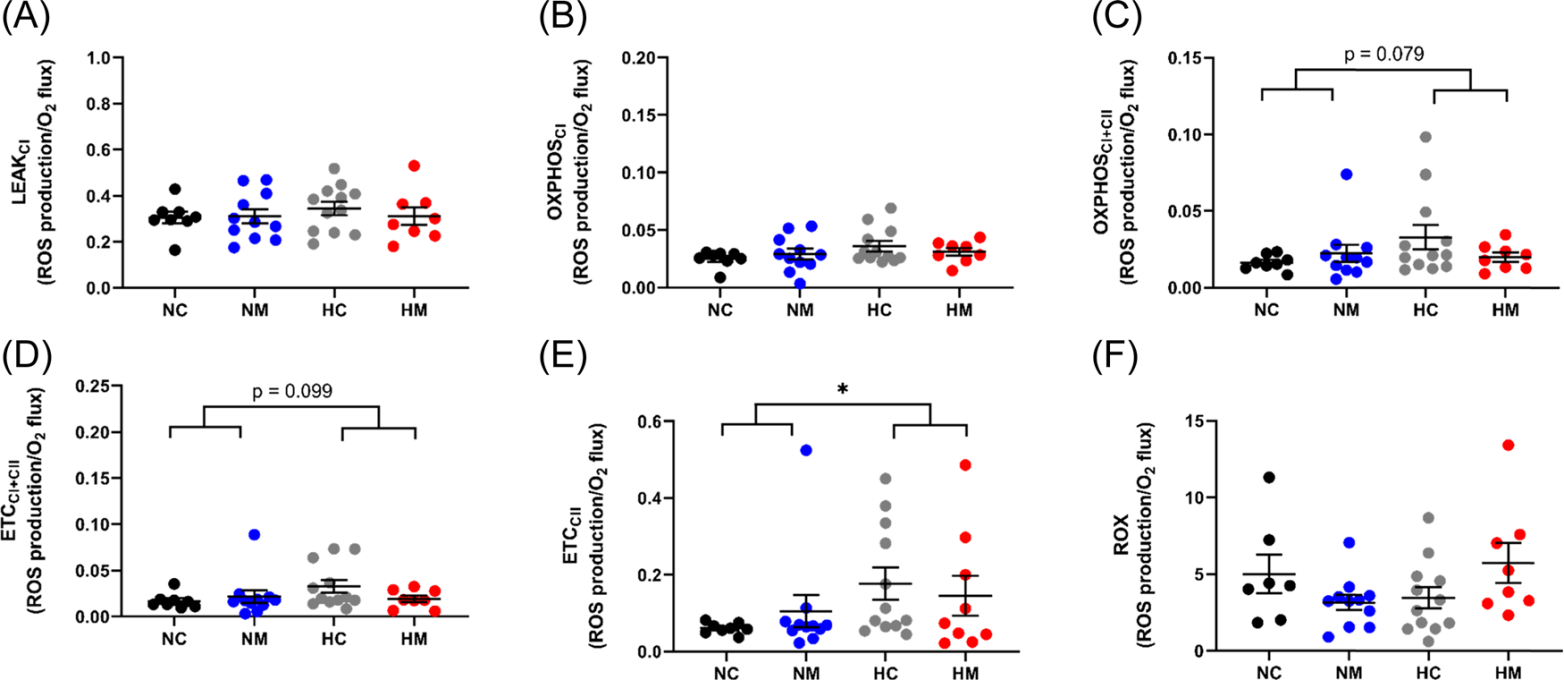
Developmental hypoxia significantly increases H_2_O_2_ production in rat heart mitochondria from male fetuses. Each panel represents a different respiratory state. *n* = 8 (NC from 6 litters), 11 (NM from 6 litters), 12 (HC from 7 litters), 8 (HM from 6 litters). *p< .05. Significance was assessed using a linear mixed model (nested). Error bars show mean ± SEM.  CI, complex I; CII, complex II; ETC, electron transport capacity; HC, hypoxia control; HM, hypoxia melatonin; NC, normoxia control; NM, normoxia melatonin; OXPHOS, oxidative phosphorylation; ROX, residual oxygen consumption; SUIT, substrate–uncoupler–inhibitor–titration.

**Figure 7:**
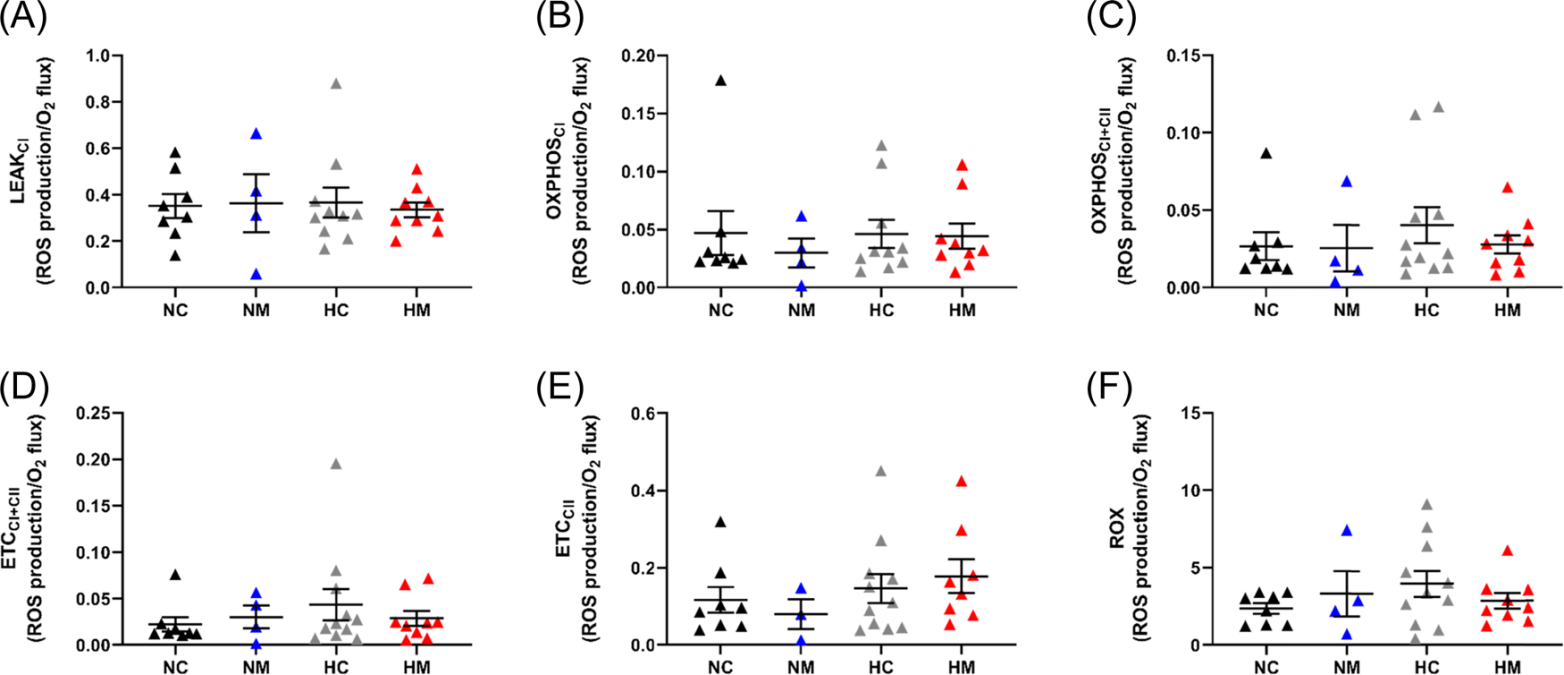
Developmental hypoxia does not change H_2_O_2_ production in rat heart mitochondria from female fetuses. Each panel represents a different respiratory state. *n* = 8 (NC from 7 litters), 4 (NM from 3 litters), 11 (HC from 8 litters), 9 (HM from 7 litters). Significance was assessed using a linear mixed model (nested). Error bars show mean ± SEM.  CI, complex I; CII, complex II; ETC, electron transport capacity; HC, hypoxia control; HM, hypoxia melatonin; NC, normoxia control; NM, normoxia melatonin; OXPHOS, oxidative phosphorylation; ROX, residual oxygen consumption; SUIT, substrate–uncoupler–inhibitor–titration.

**Figure 8:**
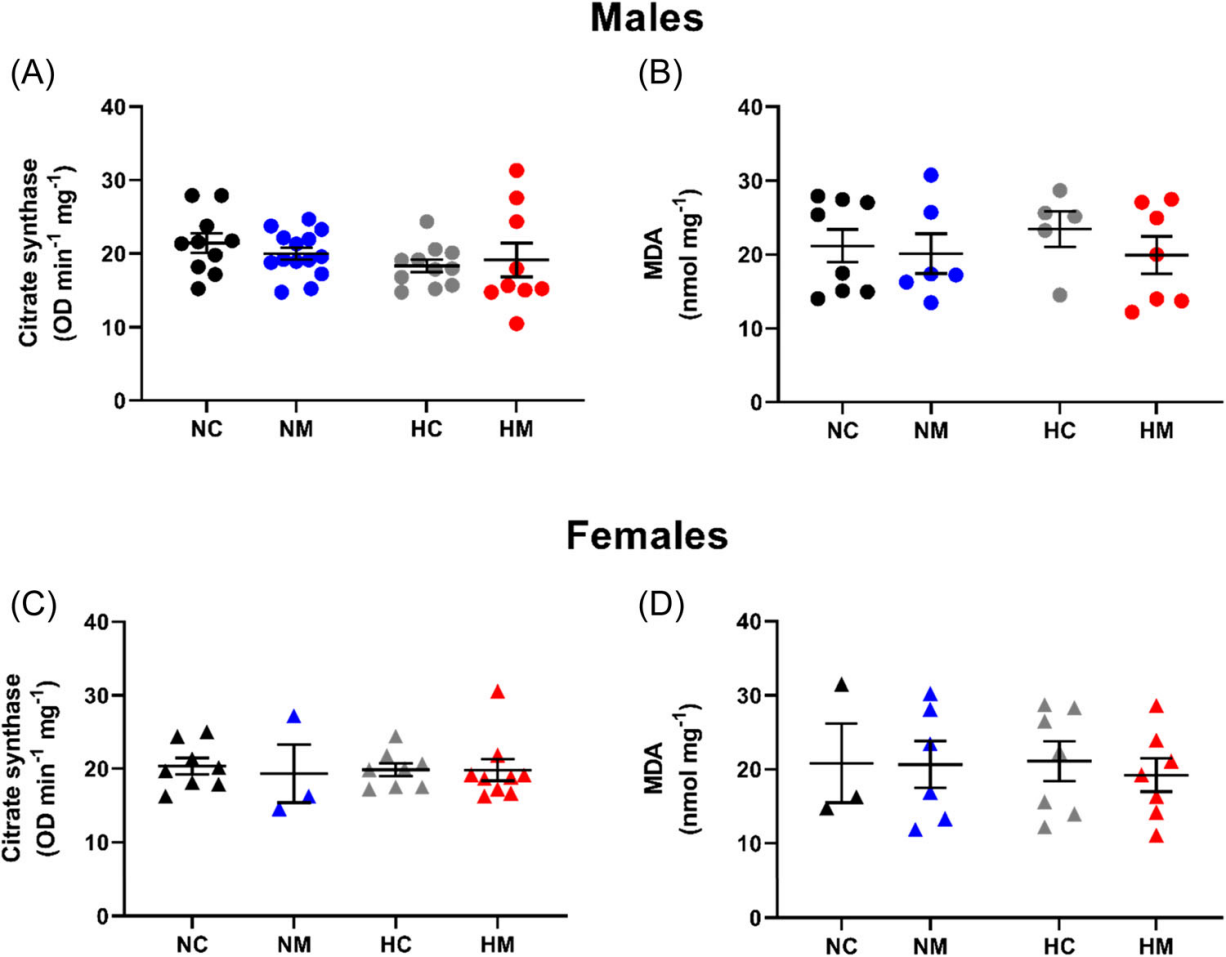
Effects of developmental hypoxia on citrate synthase activity and lipid peroxidation in fetal ventricles. Citrate synthase activity and MDA were measured in homogenized ventricular tissue from male (A, B) and female (C, D) fetuses. Circular symbols show males, triangular symbols show females. For MDA (males), *n* = 8 (NC from 6 litters), 6 (NM from 6 litters), 5 (HC from 4 litters), 7 (HM from 6 litters). For MDA (females), *n* = 3 (NC from 3 litters), 6 (NM from 5 litters), 7 (HC from 6 litters), 7 (HM from 6 litters). For citrate synthase (males), *n* = 10 (NC from 7 litters), 14 (NM from 7 litters), 11 (HC from 6 litters), 9 (HM from 6 litters). For citrate synthase (females), *n* = 8 (NC from 6 litters), 3 (NM from 3 litters), 8 (HC from 6 litters), 9 (HM from 7 litters). Significance was assessed using a linear mixed model (nested). Error bars show mean ± SEM. HC, hypoxia control; HM, hypoxia melatonin; MDA, malondialdehyde; NC, normoxia control; NM, normoxia melatonin; OD, optical density.

As mitochondrial H_2_O_2_ production is dependent on O_2_ consumption, we expressed H_2_O_2_ production normalized to O_2_ consumption and found that H_2_O_2_ production/O_2_ consumption was increased in the hypoxic male cohort (Figure 6). This effect was apparent in the OXPHOS_I + II_, ETC_I + II_, and ETC_CII_ states, but only reached significance in the ETC_CII_ state (main hypoxia effect *p* = .027) (Figure 6C–E). There were no differences in basal mitochondrial H_2_O_2_ production when normalized to protein in any of the male experimental groups (Supporting Information: Figure 2). Similar to males, there were no differences in basal mitochondrial H_2_O_2_ production normalized to protein in any of the female experimental groups (Supporting Information: Figure 3), and there were also no differences when H_2_O_2_ production was normalized to O_2_ consumption (Figure 7). To determine whether changes in male H_2_O_2_ production/O_2_ consumption led to oxidative stress, we measured lipid peroxidation (assessed with the TBARS assay) and found no differences between normoxic or hypoxic groups in either sex (Figure 8B,D). Finally, melatonin treatment had no effect on H_2_O_2_ production, H_2_O_2_ production/O_2_ consumption or lipid peroxidation in any of the groups studied (Figures 6, 7, 8). The MANOVA analysis agreed with the above findings, showing no interaction effects between the four groups (*p* = .072), but a statistically significant effect of hypoxia (*p* = .007).

## 4 DISCUSSION

This is the first study to determine the effects of developmental hypoxia and maternal melatonin treatment on mitochondrial respiration and ROS production in the fetal heart. The principle findings of this study were that hypoxia reduced mitochondrial respiratory capacity in the hearts from male, but not female, fetuses. Male cardiac mitochondria showed increased rates of H_2_O_2_ production/O_2_ consumption, but this was not associated with increased lipid peroxidation. Therefore, the data show that developmental hypoxia has sex‐specific effects on cardiac mitochondria in the fetus, which may explain the differential risk to cardiac metabolic disease in later life in male and female offspring.

### 4.1 Effects of hypoxia and maternal melatonin treatment on fetal and maternal biometry

We found that hypoxia at 13% O_2_ from Days 6 to 20 had no effect on fetal body weight, but did cause an increase in placental weight and decrease in placental efficiency, which is consistent with previous reports of hypoxic pregnancy.^7^, ^55^, ^56^ These findings reinforce the idea that in response to hypoxia in early pregnancy, placental growth is enhanced to maintain fetal growth, with the reduction in placental efficiency reflecting this.^6^, ^56^ Maternal melatonin treatment did not influence fetal or placental weight outcomes in this study.

### 4.2 Effects of hypoxia on heart mitochondrial respiratory capacity and H_2_O_2_ production in male fetuses

Male fetuses exposed to developmental hypoxia exhibited a mild but significant reduction in heart mitochondrial respiratory capacity and an increase in H_2_O_2_ production/O_2_ consumption. Importantly, we recently found a more pronounced version of the same phenotype in adult male mice from hypoxic pregnancies.^31^ Therefore, combined, our studies suggest pathways involved in mitochondrial function and ROS production can be programmed by gestational hypoxia, and the phenotype may become more pronounced with aging. These changes in mitochondrial function in the heart may contribute to the range of cardiac abnormalities that are evident in adult male offspring from hypoxic pregnancies, including diastolic dysfunction,^9^ sympathetic dominance,^6^ and sensitivity to ischemia‐reperfusion injury.^16^, ^17^ In this regard, future work should investigate the effects of gestational hypoxia to stress‐tolerance in offspring cardiac mitochondria.

A reduction in mitochondrial respiration is a common response in adult mammals^27^, ^57^ and ectothermic vertebrates^58^, ^59^, ^60^ subjected to chronic hypoxia. In particular, lowlander humans acclimatizing to hypoxia during an ascent to Mount Everest have reduced mitochondrial respiratory capacity in skeletal muscle, and similar to our study, this is only evident with CII substrates.^61^ The physiological significance of reducing respiratory capacity in hypoxia is debatable. At first sight, the response may seem counterintuitive because ATP production will be reduced when ATP demand already outstrips supply. However, a lower respiratory rate also serves to reduce electron leak from the electron transport chain, which should limit ROS production.^27^ While H_2_O_2_ production was higher in the hypoxic cohort when H_2_O_2_ was normalized to O_2_ consumption, absolute levels of H_2_O_2_ in males were similar between groups when normalized to protein content. These results support the notion that by reducing respiratory capacity in hypoxia, absolute ROS production is maintained, representing an adaptive response to limit overall ROS production. Reducing flux through CII is particularly relevant, as this enzyme is heavily implicated in the overproduction of ROS during ischemia and reperfusion injury.^62^ Nevertheless, lower respiratory capacity has negative consequences for ATP production, and while previous work suggests humans at high altitudes overcome this problem by reducing proton leak pathways to increase mitochondrial efficiency,^61^ the present study found no effects of hypoxia on leak respiration rate or coupling efficiency (RCR) in male fetuses. In addition, our study on adult male mice from hypoxic pregnancies showed the reduction in respiratory capacity persists into adulthood and worsens, which is associated with an even greater basal H_2_O_2_ production.^31^ Therefore, while mitochondrial remodeling during fetal hypoxia may be protective in the short term, it may become maladaptive in the long term.

Our experimental design also provides insight into the mechanism reducing respiratory capacity in male fetuses from hypoxic pregnancies. The lower respiratory rate was not associated with any changes in CS activity, which suggests decreased mitochondrial content is unlikely to be the underlying mechanism.^63^ However, since the reduced rate was still evident in the presence of a CI inhibitor (rotenone), and absent when electrons were donated directly to CIV, our results suggest that the mechanism is likely to involve CII and/or CIII. It is possible that protein expression of these complexes is reduced by hypoxia exposure. Indeed, previous work on a guinea pig model of chronic hypoxia in late gestation (10.5% oxygen from GD 50 for 14 days) has shown male offspring to have reduced cardiac protein expression of complexes I, III, and V.^34^, ^35^ Furthermore, our recent study on adult male mice from hypoxic pregnancies (13% oxygen from GD 6–18; term in mice is ~GD 19) showed that the reduction in respiratory capacity was associated with reduced protein expression of complexes I, II and IV.^31^ Therefore, the expression of mitochondrial proteins could be permanently modified by exposure to hypoxia, presumably via epigenetic mechanisms. In support of this concept, recent work has shown gestational hypoxia induces global changes in DNA methylation in the fetal heart, and many of the gene pathways were related to metabolism and mitochondrial function.^64^, ^65^

Our experimental design also allows us to speculate on the mechanism that underlies the increased H_2_O_2_ production in males from hypoxic pregnancies. Given that the effect was apparent when CI was inhibited with rotenone, and it disappeared when CIII was inhibited with antimycin A, our data indicate that the site of H_2_O_2_ production is the ubiquinone redox site, Qi, of CIII—the site bound by antimycin A.^66^ CIII is a major producer of ROS within the mitochondrial respiratory chain, and production of ROS at this complex is linked with a wide variety of cardiac disease models associated with hypoxia.^67^, ^68^ Nevertheless, the decrease in respiratory capacity in male fetuses likely compensates for the increased H_2_O_2_ production, which is consistent with the lack of any lipid peroxidation. It is also possible that oxidative stress was limited by increasing antioxidant capacity, as previous metabolomic research has shown the potent antioxidant bilirubin is increased in the hypoxic fetal heart.^69^ The lack of hypoxia‐induced oxidative stress contrasts with previous work where prenatal hypoxia increased markers of oxidative stress in the rat, sheep, and guinea pig heart.^35^, ^69^, ^70^ However, these studies used a more severe level of hypoxia (10%–11% O_2_), which is likely to cause greater ROS production and may also affect maternal nutrition, which could independently promote oxidative stress.^71^, ^72^ Clearly, more research is needed to unravel the effects of hypoxia on fetal ROS signaling in the heart.

### 4.3 Effects of hypoxia on mitochondrial respiratory capacity and H_2_O_2_ production in female fetuses

Additional data in the present study show that the effects of gestational hypoxia on fetal cardiac mitochondrial respiration were sex‐dependent, with females being more protected than males. Mitochondrial respiration was very similar between normoxic and hypoxic female fetuses across respiratory states, and H_2_O_2_ production was also unaffected when normalized to either protein or oxygen consumption, which is consistent with no change in lipid peroxidation. Interestingly, adult female mice from hypoxic pregnancies have increased respiratory capacity and lower H_2_O_2_ production, which was associated with the increased enzymatic activity of CIV.^31^ Therefore, fetal cardiac mitochondria in females exposed to hypoxia appear to be on a different developmental trajectory to their male counterparts. This plasticity in mitochondrial function may be one of the reasons that female adult offspring from hypoxia are less susceptible to cardiac dysfunction than males,^32^ and appear to recover better from ischemia‐reperfusion injury.^16^

Thus far, there have been limited studies investigating sex‐dependent effects of hypoxia at the fetal life stage, particularly with regard to mitochondrial function. However, one study has shown late‐onset gestational hypoxia (10.5% oxygen from GD 50 for 14 days) reduces female fetal guinea pig mitochondrial protein expression of complexes I, III, and V in the heart, but in contrast to males, this was not associated with any change in enzymatic activity of complexes IV.^34^ Interestingly, the same study showed that the timing of hypoxia was important in the guinea pig response, with early onset hypoxia (10.5% oxygen from GD 25 for 39 days) having no effects on female mitochondrial protein expression or activity. While the level of hypoxia was more severe in the guinea pig study (10.5% vs. 13% oxygen), the collective data suggest that female fetuses have a milder response to gestational hypoxia and maintain respiratory rates. These results align well with previous studies on various intrauterine stressors that show sex‐dependent differences in mitochondrial responses.^73^, ^74^, ^75^, ^76^, ^77^ One possible explanation is differential gene expression by sex‐dependent epigenetic regulation. Indeed, previous work has found differences in the expression levels of DNA methyltransferase enzymes between males and females during gestation,^78^, ^79^ and Huang et al. found sex‐dependent methylation patterns in response to prenatal hypoxia.^65^ Clearly, more work is necessary to identify the mechanisms behind sex‐dependent mitochondrial programming in the hypoxic fetus.

### 4.4 Effects of melatonin treatment

In the present study, we used melatonin as our antioxidant, because this hormone is not only safely taken by humans, but it can be transported into the mitochondria, and it is also synthesized within the mitochondrial matrix.^43^ In this way, melatonin may be considered a mitochondria‐targeted antioxidant,^44^, ^45^, ^46^ which scavenges ROS at the main site of free radical synthesis.^48^ In the present study, we show that maternal treatment with melatonin in the drinking water led to a significant increase in the maternal and fetal plasma melatonin concentration. These data confirm that melatonin crosses the placenta in rodents when administered to the maternal drinking water. Interestingly, there was a trend toward an increased maternal melatonin concentration in the hypoxic dams compared to normoxic. Similar results have been reported in the same model elsewhere,^11^ and while the underlying reason is unclear, we speculate that hypoxia could be altering metabolism, which may affect circulating melatonin concentrations. Nevertheless, the present study shows that prenatal hypoxia did not affect the fetal plasma concentrations of melatonin.

The final concentration of melatonin in maternal and fetal plasma following melatonin treatment was similar to that measured in other studies using pregnant rats,^10^, ^80^, ^81^ and is within the range or lower than plasma concentrations in men and women following melatonin intake to avoid jet lag.^82^ However, maternal melatonin treatment had no effects on respiratory capacity, H_2_O_2_ production, or lipid peroxidation in fetal hearts in normoxic or hypoxic pregnancies. It is possible that melatonin could have a protective effect on mitochondrial pathways and properties that were not measured in this study, such as the permeability transition pore, DNA mutations, membrane stability, or mitochondrial dynamics.^43^ Furthermore, our study only investigated the chronic effects of fetal hypoxia on mitochondrial respiration and ROS production. Acute reoxygenation of heart tissue poses a significant challenge to hypoxic fetuses, and therefore, melatonin may have shown greater protective effects against cellular injury if measured at the reperfusion phase. Nevertheless, our study suggests that maternal melatonin treatment in normoxic pregnancies does not have any negative effects on mitochondrial respiration or ROS production in the fetal heart. This is important information for developing this antioxidant as a safe intervention for pregnancy complications, particularly as melatonin is currently being trialed in human pregnancies complicated by fetal growth restriction and pre‐eclampsia.^46^, ^49^

### 4.5 Study limitations

There are some limitations related to our study design and protocol. First, the relatively small sample size may have limited the power of our study, particularly in terms of revealing the melatonin interaction effects. Indeed, there were often trends within our data that melatonin was preventing mitochondrial remodeling, and larger sample size may have resolved these effects. In addition, although we chose a dose of melatonin that was clinically relevant^42^ and previously shown to be protective,^11^ the concentration may not have been high enough to achieve metabolic protection. With respect to respiration measurements, we provided OXPHOS substrates at a saturating concentration, and therefore our results do not account for potential differences in substrate availability and/or preference in vivo. Furthermore, any posttranslational modifications, such as nitrosylation of the electron transport chain, would have been lost as a result of the homogenization process, and therefore any potential effects would not have been detected. Finally, our model of hypoxia caused a reduction in maternal weight gain, which may have had independent stress on the developing fetus even in the absence of overt growth restriction.

## 5 CONCLUSION AND PERSPECTIVES

Our study shows that developmental hypoxia has significant sex‐dependent effects on fetal cardiac mitochondrial respiration and ROS production, which may have long‐term implications for cardiac metabolic health and disease. These findings strongly align with the emerging evidence that mitochondria represent a key cellular target that underpins developmental programming (reviewed in Gyllenhammer et al.^83^). Indeed, multiple studies have demonstrated mitochondrial changes induced by intrauterine stress can persist into adulthood,^84^, ^85^, ^86^, ^87^, ^88^, ^89^, ^90^, ^91^, ^92^ and even across generations.^93^, ^94^, ^95^ Given the crucial relationship between mitochondrial function and cardiac health, intrauterine mitochondrial programming could be an important mechanism that drives cardiovascular disease susceptibility in adulthood. Our study also shows that maternal melatonin treatment has no effects on fetal cardiac mitochondrial respiration and ROS production, despite its ability to prevent cardiac programming in adult offspring of hypoxic pregnancies.^11^ The fact that melatonin had no negative effects on mitochondrial parameters in normoxic pregnancies is encouraging and provides support for this antioxidant being used therapeutically in complicated pregnancies.

## AUTHOR CONTRIBUTIONS

Kerri L. M. Smith, Agnieszka Swiderska, Gina L. J. Galli, Mitchell C. Lock, Lucia Graham, Wulan Iswari, Tashi Choudhary, Donna Thomas, Elizabeth C. Cottrell, and Hager M. Kowash all contributed to data acquisition and analysis. Michelle Desforges, Elizabeth C. Cottrell, Andrew W. Trafford, Dino A. Giussani, and Gina L. J. Galli contributed to the concept and experimental design. Kerri L. M. Smith, Agnieszka Swiderska, Mitchell C. Lock, Michelle Desforges, Elizabeth C. Cottrell, Andrew W. Trafford, Dino A. Giussani, and Gina L. J. Galli contributed to drafting and approval of the manuscript.

## Supporting information

Supplementary information.

## Data Availability

The data that support the findings of this study are available from the corresponding author upon reasonable request.
