# Supplementary material for: Chronic developmental hypoxia alters mitochondrial oxidative capacity and reactive oxygen species production in the fetal rat heart in a sex‐dependent manner
Source: J Pineal Res. 2022 Aug 17;73(3):e12821. doi: 10.1111/jpi.12821 (PMC9540814; doi:10.1111/jpi.12821)
Supplement: Supplementary file 1 — Supplementary information. [file JPI-73-e12821-s001.docx]

**
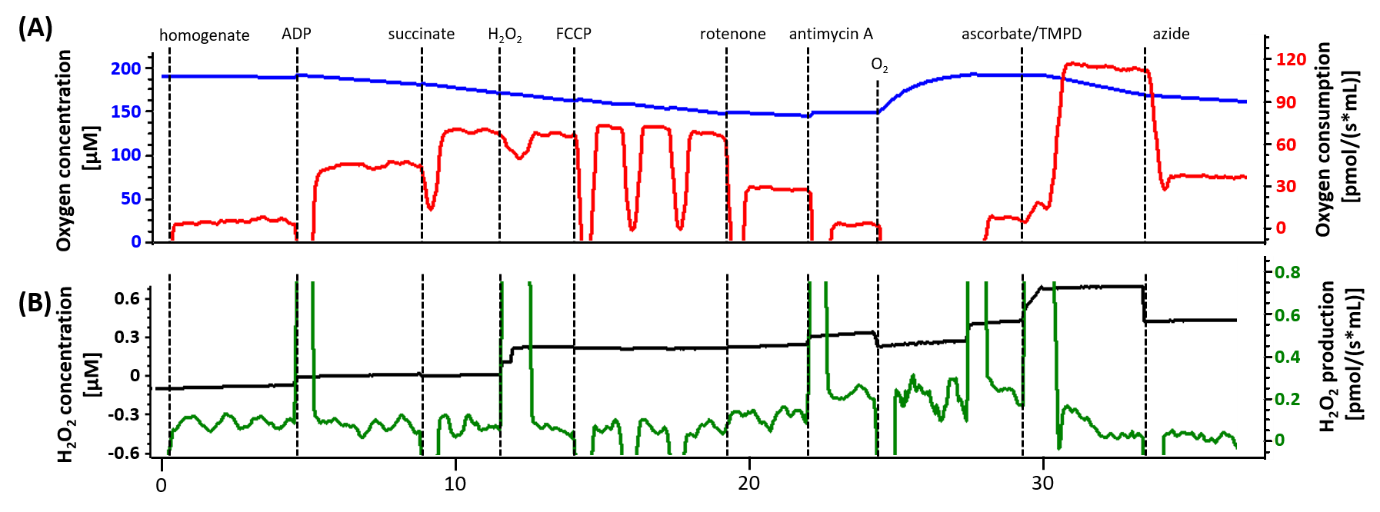
Supplementary**


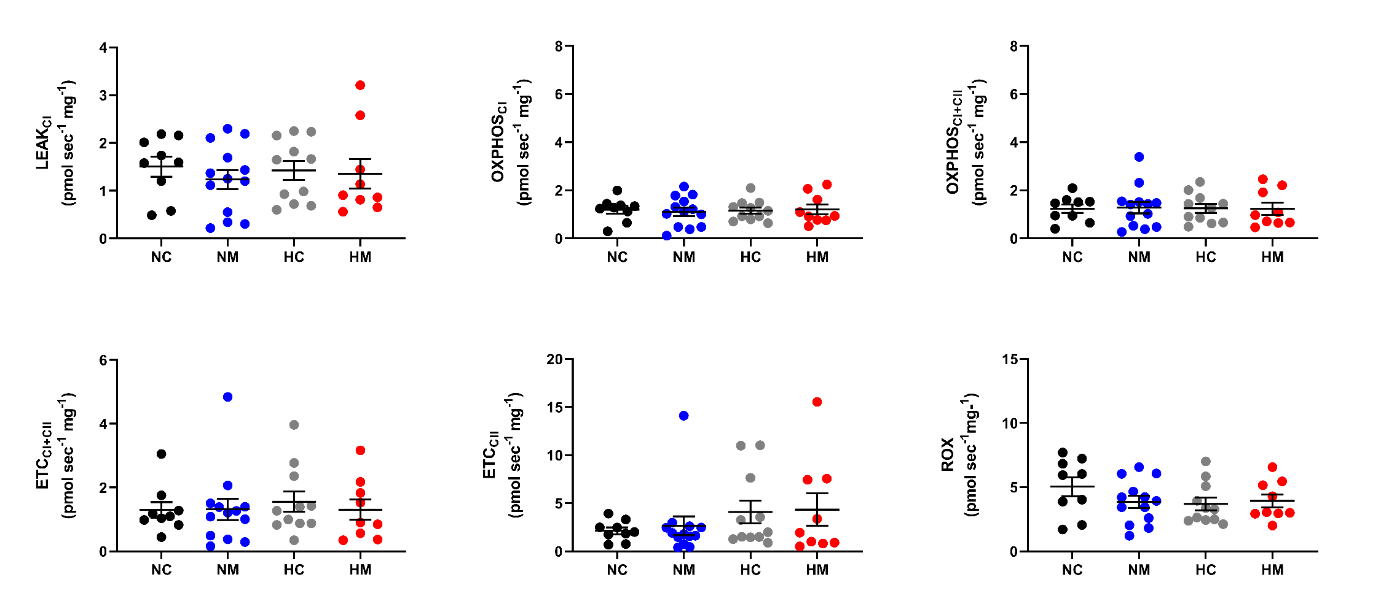


**Supplementary Figure 1. Original trace of mitochondrial oxygen consumption with simultaneous hydrogen peroxide (H_2_O_2_) production.** Mitochondrial oxygen consumption (A) and H_2_O_2_ production (B) trace from a normoxic control fetal rat (female, ventricular homogenate). Prior to homogenate injection, malate, pyruvate and glutamate were added as substrates for complex I. Additionally, horseradish peroxidase, Amplex UltraRed and superoxide dismutase were added in order to measure H_2_O_2_ production. Compounds were added in sequence to measure oxygen consumption and H_2_O_2_ production at various complexes of the electron transport chain. *Some chemicals caused injection artefacts in the H_2_O_2_ trace; these were not used for analysis (measurements were taken from a stable region of the trace)*. *ADP = adenosine diphosphate, FCCP = carbonyl cyanide-4-(trifluoromethoxy)phenylhydrazone, TMPD = N,N,N,N-tetramethyl-p-phenylenediamine.*

**Supplementary Figure 2. Effects of developmental hypoxia on H_2_O_2_ production in rat heart mitochondria from male fetuses.** Each panel represents a different respiratory state. *NC = normoxia control, NM = normoxia melatonin, HC = hypoxia control, HM = hypoxia melatonin, OXPHOS = oxidative phosphorylation, CI = complex I, CII = complex II, CIV = complex IV, ETC = electron transport capacity, ROX = residual oxygen consumption. n = 9 (NC from 6 litters), 13 (NM from 6 litters), 12 (HC from 7 litters), 9 (HM from 6 litters). Significance was assessed using a linear mixed model (nested). Error bars show mean ± SEM.*


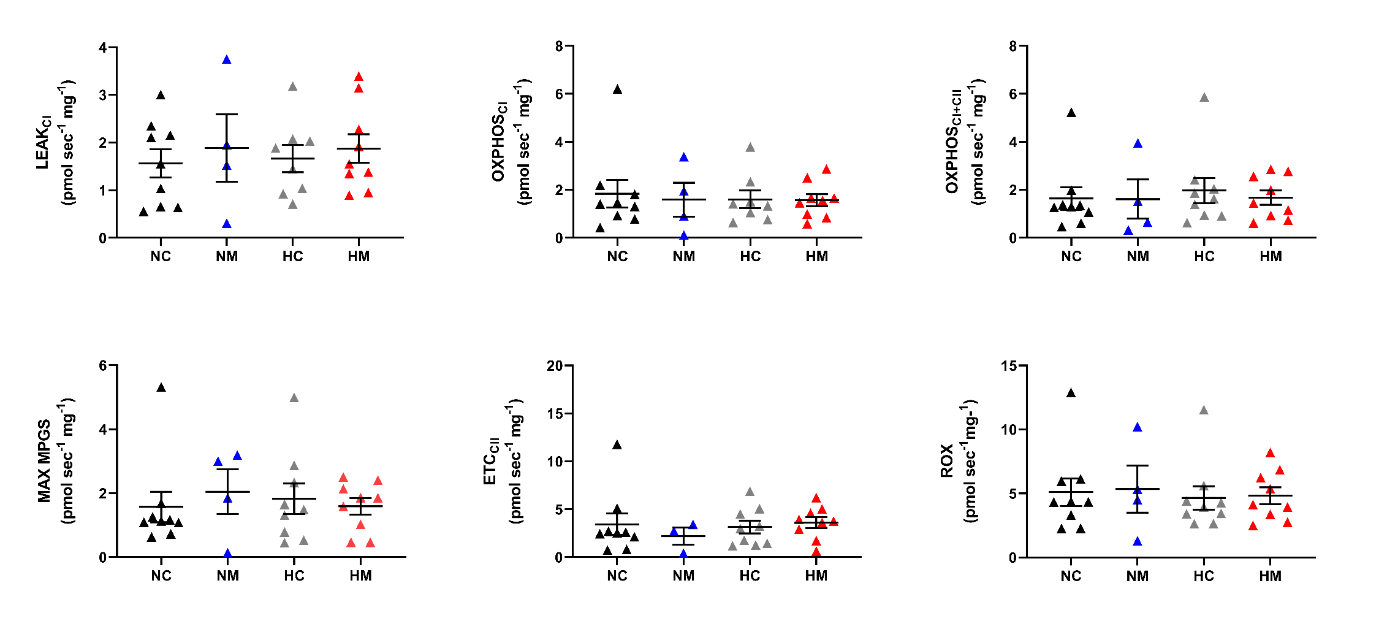


**Supplementary Figure 3. Effects of developmental hypoxia on H_2_O_2_ production in rat heart mitochondria from female fetuses.** Each panel represents a different respiratory state. *NC = normoxia control, NM = normoxia melatonin, HC = hypoxia control, HM = hypoxia melatonin, OXPHOS = oxidative phosphorylation, CI = complex I, CII = complex II, CIV = complex IV, ETC = electron transport capacity, ROX = residual oxygen consumption. n = 9 (NC from 7 litters), 4 (NM from 3 litters), 11 (HC from 8 litters), 9 (HM from 7 litters). Significance was assessed using a linear mixed model (nested). Error bars show mean ± SEM.*
